# PRESERFLO ™ MicroShunt versus trabeculectomy: 1-year results on efficacy and safety

**DOI:** 10.1007/s00417-023-06075-4

**Published:** 2023-05-03

**Authors:** Melanie Jamke, Robert Herber, Maike A. Haase, Carolin S. Jasper, Lutz E. Pillunat, Karin R. Pillunat

**Affiliations:** Universitätsklinikum Carl Gustav Carus an der Technischen Universität Dresden, Fetscherstrasse 74, 01307 Dresden, Germany

**Keywords:** PRESERFLO™ MicroShunt, MicroShunt, Trabeculectomy, Open-angle glaucoma, 24-h IOP, Mean diurnal IOP, Peak diurnal IOP, Diurnal IOP fluctuation

## Abstract

**Purpose:**

To compare the efficacy and safety of the PRESERFLO™ MicroShunt versus trabeculectomy in patients with primary open-angle glaucoma (POAG) after one year.

**Patients and methods:**

Institutional prospective interventional cohort study comparing eyes with POAG, which had received the PRESERFLO™ MicroShunt versus trabeculectomy. The MicroShunt group was matched with the trabeculectomy group for age, known duration of disease, and number and classes of intraocular pressure (IOP) lowering medications to have similar conjunctival conditions. The study is part of the Dresden Glaucoma and Treatment Study, using a uniform study design, with the same inclusion and exclusion criteria, follow-ups and standardized definitions of success and failure for both procedures. Primary outcome measures: mean diurnal IOP (mdIOP, mean of 6 measurements), peak IOP, and IOP fluctuations. Secondary outcome measures: success rates, number of IOP lowering medications, visual acuity, visual fields, complications, surgical interventions, and adverse events.

**Results:**

Sixty eyes of 60 patients, 30 in each group, were analyzed after 1-year follow-ups. Median [Q25, Q75] mdIOP (mmHg) dropped from 16.2 [13.8–21.5] to 10.5 [8.9–13.5] in the MicroShunt and from 17.6 [15.6–24.0] to 11.1 [9.5–12.3] in the trabeculectomy group, both without glaucoma medications. Reduction of mdIOP (P = .596), peak IOP (P = .702), and IOP fluctuations (P = .528) was not statistically significantly different between groups. The rate of interventions was statistically significantly higher in the trabeculectomy group, especially in the early postoperative period (P = .018). None of the patients experienced severe adverse events.

**Conclusion:**

Both procedures are equally effective and safe in lowering mdIOP, peak IOP and IOP fluctuations in patients with POAG, one year after surgery.

Clinical trial registration: NCT02959242.


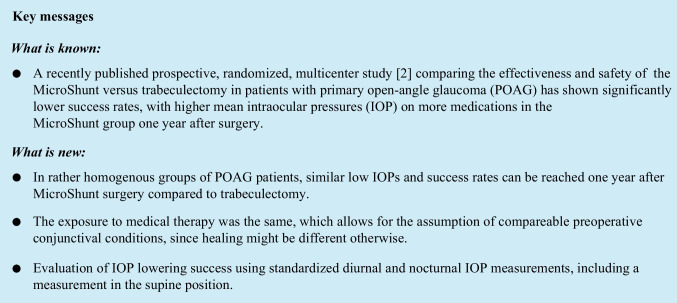


## Introduction

Glaucoma is one of the most common causes of irreversible blindness worldwide with increasing prevalence. It is estimated that the global prevalence of glaucoma will affect approximately 111.8 million people by 2040 [3].

Glaucoma progression can be delayed or prevented by reducing intraocular pressure (IOP). Moreover, IOP is the only risk factor that can be significantly influenced with IOP-lowering medication, laser treatments or surgery. Until now, trabeculectomy has been considered to be the gold standard of glaucoma surgery for moderate to severe glaucoma cases [4]. In this regard, although trabeculectomy has proven to be a good IOP-lowering surgical option, it is also known that it can be associated with time consuming postoperative care, which delays recovery, and sometimes severe, potentially sight-threatening complications [5, 6]. In this context, interest in minimally invasive glaucoma surgery (MIGS) has increased in recent years and has been the subject of numerous studies and reviews with the prospect of lower complication rates, shorter surgical time and less postoperative follow-up [7–9]. Since IOP reduction is often quite modest [10], MIGS are only indicated in glaucoma patients with mild-to-moderate disease.

One of these possible alternatives is the novel PRESERFLO™ MicroShunt (Santen Inc., Osaka, Japan) which is regarded as a less invasive glaucoma surgery (LIGS). It is an ab-externo shunt device with a length of 8.5 mm, an external diameter of 350 µm and a lumen diameter of 70 µm. This design limits flow and filtrates aqueous humor from the anterior chamber continuously and more posteriorly to the subtenon space forming a posterior bleb. Of importance is the highly biocompatible and bioinert material used for production: poly (-styrene-block-isobutylene-block-styrene) (SIBS) [11]. This material is also used for the coating on the TAXUS® drug-eluting coronary stent (Boston Scientific Corp., MA, USA) [12]. The advantages of SIBS used in the PRESERFLO™ MicroShunt are its high biocompatibility which reduces the local inflammatory response resulting in less risk of encapsulation and conjunctival scarring [13].

In addition to the material, the formation of a posterior filtering bleb with potentially lower risks of long-term complications like blebitis and bleb-related endophthalmitis is a further advantage compared to trabeculectomy with a more anterior filtering bleb close to the limbus [14]. Furthermore, the procedure is considered to be less invasive and with a faster recovery.

The shunt has been approved in Europe (Conformité Européenne – CE) since 2012 and has been easily available since 2019.

Many studies have already demonstrated the effective and safe IOP-lowering with substantial reduction of anti-glaucoma medication after PRESERFLO™ Micro-Shunt implantation in primary open-angle (POAG) [15–17], pseudoexfoliation glaucoma patients [18, 19], and glaucoma refractory to previous subconjunctival surgery [20]. To date though, there are only few studies available comparing the efficacy and safety of the PRESERFLO™ Micro-Shunt with trabeculectomy [2, 17, 21].

Initial comparisons in rabbit eyes yielded positive results in terms of shorter operative times with the Micro-Shunt and an equivalent pressure-lowering effect compared to trabeculectomy [22].

Following the 6-month results, which have demonstrated an equivalent IOP lowering effect of the PRESERFLO™ MicroShunt with fewer follow-up visits and postoperative procedures in the early postoperative phase compared to trabeculectomy [17], the current study compares the effectiveness and safety of stand-alone mitomycin C-augmented (MMC) PRESERFLO™ MicroShunt implantation versus MMC-augmented trabeculectomy in patients with POAG after the first year.

## Materials and methods

### Study design

Single center prospective interventional cohort study evaluating the efficacy, success rates, and safety of primary stand-alone MMC-augmented PRESERFLO™ MicroShunt implantation compared with primary stand-alone MMC-augmented trabeculectomy in patients with POAG not achieving adequate IOP-reduction on maximally tolerated IOP-lowering medication. Although randomization was not performed, both groups were subject to a consistent study design, which followed equal inclusion and exclusion criteria, follow-ups, and standardized definitions of success and failure. The study is part of the Dresden Glaucoma and Treatment Study (DGTS; http://www.clinicaltrials.gov; NCT02959242).

The study protocol follows the tenets of the Declaration of Helsinki and was approved by the Institutional Review Board of the Medical Faculty Carl Gustav Carus of the Technische Universität Dresden, Germany (EK 43310–2015). All participants agreed to take part in the study and signed a written informed consent. No funding was received for conducting this study.

### Patient selection

Since June 2019, when the PRESERFLO™ MicroShunt was easily available in Europe, all patients with POAG with the need for glaucoma filtering surgery were offered the novel MicroShunt and included if inclusion and exclusion criteria (see below) were met. Reasons for decision to surgery were insufficient IOP-control on maximum tolerated IOP-lowering medication, visual field progression, poor adherence or intolerance to topical medication with topical and/or systemic side effects. Patients were consecutively included and analyzed if they had a minimum of a 1-year follow-up up to December 2021. In the case of both eyes being eligible, one was randomly selected. The trabeculectomy group received MMC-augmented filtering surgery for the same reasons as described above. Groups were matched for age, duration of application, number and classes of IOP-lowering medications to have a similar conjunctival condition as close as possible, which is essential for any bleb-forming filtering glaucoma surgery. Besides this, there was no other evaluation of the conjunctival condition like grading hyperemia etc. The inclusion period of the trabeculectomy group was from 2016 to 2019. Patients in both groups were of White/European ethnicity.

POAG included high (HPG; untreated IOP > 21 mmHg) and normal pressure glaucoma (NPG; untreated IOP ≤ 21 mmHg) patients. They showed typical cupping of the optic nerve head, thinning of the neuroretinal rim and damage to the inner layers of the retina on optical coherence tomography (OCT), corresponding visual field defects (without any other ocular or systemic causes for these defects), and an open angle on gonioscopy. Further inclusion criteria were eyes with an axial length ≥ 22 mm, an anterior chamber (AC) volume > 110 mm^3^, and an AC depth of > 2.1 mm. Eligible patients were aged more than 40 years at the time of their first diagnosis. Exclusion criteria were previous ophthalmic surgeries, except for uncomplicated phacoemulsification or selective laser trabeculoplasty 3 months prior to study inclusion. Eyes with endothelial cell densities below 1000 cells/mm^2^ as well as training cases in the MicroShunt group were excluded.

### Baseline and follow-up examinations

Baseline recordings were taken preoperatively at the time when the decision for surgery was made and included age, gender, and known duration of the disease, number and classes of IOP lowering medications as well as previous surgeries. Follow-ups took place weekly during the first postoperative month, at 6 and at 12 months. Baseline, 6- and 12-month examinations included refraction, best corrected visual acuity (BCVA), a thorough examination of the anterior and posterior segment, gonioscopy, a full glaucoma work-up, and a day-night IOP profile using Goldmann applanation tonometry (GAT). Measurements were taken at 1, 4, 7 and 10 pm at the slitlamp (Haag-Streit, Koeniz, Switzerland), at midnight in a supine position with a handheld Perkins MK3 tonometer (HS Clement Clark Ophthalmic, Haag.Streit UK), and at 7 am, again at the slitlamp. At each time-point (± 0.5 h), a masked observer took one measurement. Automated perimetry was performed using the Humphrey field analyzer (Swedish interactive threshold algorithm standard 30–2 program; Carl Zeiss Meditec. Dublin, CA, USA). Visual field (VF) damage was considered as early with a mean deviation (MD) ≥ -6 dB, moderate with a MD between -6 dB and -12 dB, and advanced with a MD ≤ -12 dB. Any VF test location that had 10 dB or less from an age-matched value caused by glaucoma affecting either or both points closest to the point of fixation and at either or both of the corresponding test points in the lower hemifield was considered as threatening fixation [23]. All of the patients were experienced in visual field testing. A complete glaucoma work-up included confocal scanning laser ophthalmoscopy (HRT II, Heidelberg Engineering Inc., Heidelberg, Germany), OCT glaucoma module (SPECTRALIS®, Heidelberg Engineering Inc., Heidelberg, Germany) and scanning laser polarimetry (Nerve Fibre Analyzer GDxPRO, Carl Zeiss Meditec, Dublin, CA, USA). Axial length was measured with optical biometry (IOL-Master, Carl Zeiss Meditec AG, Jena, Germany) and lens thickness (Echograph B-scan-Cinescan S, Quantel Medical, Clermont-Ferraud, France), if applicable, with ultrasound. The Pentacam HR 3 (Oculus, Wetzlar, Germany) was used for objective analysis of the anterior segment and central corneal thickness. Endothelial cell density (ECD) was assessed with the CEM-530 specular microscope (NIDEK CO., LTD, Gamagori, Japan). A mean of 3 measurements was used for analyses. Any IOP-lowering substances and interventions necessary were recorded.

Baseline and follow-up examinations were performed by different masked observers.

### Surgical procedures

Both procedures were performed by either of 2 experienced glaucoma surgeons (K.R.P. and L.E.P.) under general anesthesia with the following standardized techniques [17].

To optimize the conjunctival condition, topical IOP-lowering therapy was stopped and switched to dexamethasone 3 times daily and systemic IOP-lowering with acetazolamide 10 days before surgery. This was the case only once in the trabeculectomy and twice in the MicroShunt group (p = 0,495, Chi^2^ test). In the case of a patent filtering surgery on the other eye or intolerance to systemic acetazolamide, topical IOP-lowering medication was continued with additional application of dexamethasone 3 times daily, 10 days before surgery. This was the case in 14 trabeculectomy and 15 MicroShunt eyes (p = 0,127, Chi^2^ test). 15 patients in the trabeculectomy and 13 patients in the MicroShunt group did not interrupt IOP lowering medications nor used dexamethasone before surgery since the procedure was carried out immediately. A complete washout of glaucoma medications was not performed since most cases had advanced stages of disease. Any oral anticoagulant or platelet aggregation inhibitor was discontinued at least 10 days before surgery after consent from the attending physician.

#### PRESERFLO™ MicroShunt (Santen Inc., Osaka, Japan)

After placing a corneal traction suture, a 3 clock-hour fornix-based conjunctival/Tenon´s flap in the superotemporal quadrant forming a wide and deep posterior pocket was created. Application of 3 corneal light shields (BVI Visitec®, Waltham, MA 02,451 USA), each 8 mm in diameter and soaked with 0.2 mg/ml (0.02%) MMC, were placed onto the bare scleral surface into this pocket for 3 min. Immediately afterwards thorough irrigation with 10 ml of balanced salt solution (BSS). Application of Miochol®-E (Acetylcholinchlorid; Dr. Mann Pharma GmbH, Germany) into the anterior chamber (AC) to straighten the iris, minimize the diameter of the pupil and to deepen the AC via a paracentesis at 8 o´clock in right and 11 o´clock in left eyes. Marking the sclera 3 mm from the limbus at the 11 o´clock position in right and the 1 o´clock position in left eyes. At the marked point preparation of a scleral pocket with the included angled triangular MANI® knife (MANI Inc., Japan) and then a needle tract into the AC with an angled 25-gauge needle. Insertion of the MicroShunt into the AC via the needle tract, tucking the fin of the device into the scleral pocket. Priming the MicroShunt at the distal end with BSS via a 23-gauge cannula and checking for continuous drop formation out of the distal end. Repositioning of the Tenon and the conjunctiva towards the limbus, avoiding any occlusion at the distal end of the device. 10–0 nylon (Ethilon™, Ethicon® LLC; Guaynabo, Puerto Rico, USA) single-knot sutures at the limbus and as needed. If the Tenon was too tight to be positioned towards the limbus it was sutured separately to the sclera with 9–0 absorbable sutures (Marlin®; Catgut GmbH, Germany).

#### Trabeculectomy

Preparation of a 2 to 3 clock-hour fornix-based conjunctival/Tenon´s flap in the superior quadrant of the eye. Before creating a 3 × 3 mm partial thickness scleral flap, application of two 9 × 4 mm Merocel® sponges soaked with 0.2 mg/ml (0.02%) MMC for 3 min onto the bare sclera. Thorough irrigation with 10 ml BSS. Filling the AC with 1.4% sodium hyaluronate (Protectalon 1.4%, VSY Biotechnology, Amsterdam, The Netherlands) via a paracentesis at 10 o`clock to avoid a rapid IOP drop after sclerostomy with the Kelly punch. Creating a peripheral iridectomy via the sclerostomy. Suturing the scleral flap with 2 and the Tenon and conjunctiva with 2–3 single-knot 10–0 nylon sutures. Application of BSS into the AC to elevate the filtering bleb and watch for bleb leaks. A 10–0 nylon mattress suture was set at the limbus to prevent postoperative leakage.

After both procedures patients received an intracameral injection of 3 mg Bevacizumab (Avastin®, Roche, USA) [24] and a combination of gentamycin and dexamethasone subconjunctivally into the lower conjunctival fold.

### Postoperative management

Postoperative treatment was standardized with preservative-free topical steroids (Dexamethasone; Dexa EDO®, Dr. Mann Pharma GmbH, Germany) 5 times a day for 4 weeks, with gradual tapering thereafter and treatment with preservative-free topical antibiotics (Ofloxacin; Floxal EDO®, Dr. Mann Pharma GmbH, Germany) 3 times a day for a week. A preservative-free mydriatic (Cyclopentolat; Zyklolat EDO®, Dr. Mann Pharma GmbH, Germany) was applied twice a day for a week in the trabeculectomy group. Anterior chamber reformation, bleb needling, and laser suture lysis were performed as needed [17]. If IOP-lowering medication was reintroduced, it was usually at the discretion of the general ophthalmologist.

### Outcome measures

Primary outcome measures were mean diurnal IOP (mdIOP, mean of 6 measurements including one measurement in a supine position), peak diurnal IOP, and diurnal IOP fluctuations one year after surgery. Secondary outcome measures included success rates, use of glaucoma medical therapy, BCVA, VF and ECD, as well as complications and surgical interventions necessary, and adverse events.

As detailed in our previous study [17] complete success was defined as mdIOP and peak diurnal IOP a) ≤ 18 mmHg for cases with early glaucoma and without threat of fixation and b) mdIOP ≤ 14 mmHg and peak IOP ≤ 18 mmHg [25] for cases with early glaucoma (MD < -6 dB) and with threat of fixation, moderate and advanced cases. Both without hypotony (i.e. IOP ≤ 5 mmHg) and without the need of any IOP-lowering medication. Qualified success was defined with the same criteria but allowed for IOP-lowering medication. Overall success consists of both complete and qualified success. A mdIOP and peak diurnal IOP higher than a) 18 mmHg for cases with early glaucoma without threat of fixation and b) mdIOP > 14 mmHg and peak IOP > 18 mmHg for cases with early glaucoma with threat of fixation, moderate and advanced cases or hypotony (i.e. IOP ≤ 5 mmHg) were considered pressure failures. Necessary surgical revisions, secondary IOP-lowering interventions, or the occurrence of severe adverse events such as loss of light perception accounted for surgical or complete failures. In the case of reoperation for glaucoma, data were censored at the time of the second glaucoma operation. Reformation of the AC or bleb needling were not considered as reoperation or as failure [2, 15, 16, 20, 26].

Since most patients were advanced in the disease a preoperative medication wash-out was not carried out, and medicated preoperative mdIOP was already relatively “low” in many cases. Defining success or failure attaining a prespecified percentage reduction of IOP did not seem appropriate and may be more difficult to achieve.

### Statistical analysis

Sample size calculations were based on the IOP reduction of the *Primary Tube Versus Trabeculectomy (PTVT) Study* [27]. A sample size of at least 4 patients per group (alpha = 0.05; power = 0.80) was required (G Power 3.1.9.2. sample size software; University of Duesseldorf, Germany). Due to the unknown dropout rate of the novel implant, we decided to include more than 20 patients per group.

Data analysis was performed using the SPSS software (version 25, IBM Statistics; New York, USA). Normal distribution was verified with the Kolmogorov–Smirnov test and Q-Q plots. The majority of primary and secondary outcome variables showed a non-normal distribution. Therefore, non-parametric tests such as the Mann–Whitney *U* test (group comparisons) and Wilcoxon test (longitudinal samples) were used for continuous variables (e.g., IOP). Results were presented as median and interquartile range [IQR, Q25 – Q75]. To analyze dichotomous variables, Fisher’s exact test or Chi^2^ test were performed. Absolute IOP reduction represents the amount of IOP reduction in mmHg and relative IOP reduction in percent compared to baseline. Patients who required any surgical revision were censored from analyses from the date of reoperation. A two-sided p-value lower than 0.05 was considered statistically significant.

## Results

### Baseline characteristics

A total of 60 eyes, 30 in each group, were included in the analysis. Table 1 provides baseline and demographic data of both groups. The groups were matched for age (p = 0.44, Mann–Whitney *U* test), the known duration of disease (p = 0.61, Mann–Whitney *U* test) and the preoperative number (p = 0.34, Mann–Whitney *U* test) and classes of IOP-lowering medication. In addition, the use of preservative free IOP-lowering medications was not statistically significantly different between the groups (p = 0.621, Chi^2^ test). Baseline mdIOP (p = 0.32, Mann–Whitney *U* test), peak diurnal IOP (p = 0.36, Mann–Whitney *U* test), and diurnal IOP fluctuations (p = 0.64, Mann–Whitney *U* test) were not different between groups.

**Table 1:** Demographics and baseline characteristics

	PRESERFLO™ MicroShunt (n = 30)	Trabecuectomy (n = 30)	*p* value
age [yrs]	68.0 [62.8—79.0]	68.5 [61.0—77.0]	0.441*
Gender: f/m; no. (%)	15 (50) / 15 (50)	14 (47) / 16 (53)	0.796^#^
Eyes: r/l; no. (%)	14 (47) / 16 (53)	19 (63) / 11 (37)	0.194^#^
md IOP [mmHg]	16.3 [13.8—21.2]	17.6 [15.6—24.0]	0.315*
md IOP fluct. [mmHg]	10.0 [6.0—12.3]	8.5 [5.0—13.3]	0.640*
peak IOP [mmHg]	22.0 [17.0—27.3]	23.5 [20.0—30.0]	0.359*
no. IOP-lowering medications	4.0 [3.0—4.0]	4.0 [3.8—4.0]	0.340*
- Prostaglandins (%)	27 (90)	30 (100)	0.237^+^
- ß‐blocker (%)	21 (70)	25 (83)	0.360^+^
- Alpha‐2‐agonists (%)	24 (80)	20 (67)	0.381^+^
- Carboanhydrase inhibitors (%)	24 (80)	26 (87)	0.731^+^
- Pilocarpine	7 (23)	6 (20)	1.0^+^
BCVA [LogMar]	0.1 [0.0—0.2]	0.2 [0.0—0.3]	0.027*
Axial length [mm]	23.9 [23.5—24.4]	23.9 [23.1—25.3]	0.848*
AC depth [mm]	3.8 [2.8—4.3]	2.9 [2.7—4.0]	0.102*
AC volume [mm^2^]	180.5 [154.5—198.3]	155.0 [137.8—183.3]	0.019*
Mean deviation [dB]	-7.3 [-12.4 – (-2.9)]	-9.1 [-19.2 – (-6.5)]	0.055*
- Early up to -6 dB. no. (%) With / without thread of fixation	14 (47) 3 / 11	6 (20) 1 / 5	0.090^#^
- Moderate -6 to -12 dB. no. (%)	7 (23)	11 (37)	
- Advanced worse than -12 dB. no. (%)	9 (30)	13 (43)	
Type of glaucoma			
- HPG. no. (%)	21 (70)	27 (90)	0.053^#^
- NPG. no. (%)	9 (30)	3 (10)	
Pseudophacic. no. (%)	18 (60)	11 (37)	0.120^+^
History of SLT. no. (%)	22 (73)	20 (67)	0.789^+^
Known duration of disease [yrs]	9.0 [5.0—22.3]	12.5 [5.0—24.3]	0.609*
Endothelial cell density [cell/mm^2^]	2392 [2145 – 2741]	2149 [1880 – 2365]	0.030*

yrs = years; f/m = female/male; no. = number; % = percentage; r/l = right eye/left eye; mdIOP = mean diurnal intraocular pressure; CAI = carbonic anhydrase inhibitors; BCVA = best corrected visual acuity; AC = anterior chamber; HPG = high pressure glaucoma; NPG = normal pressure glaucoma; SLT = selective laser trabeculoplasty

Continuous parameters/variables are presented as median and interquartile range [IQR, Q25-Q75]

^*^ Mann–Whitney-U test; ^#^ Chi^2^ test; ^+^ Fisher’s exact test. Significance is marked in bold (*p* < 0.05)

### Primary outcome measures

**Fig. 1:**
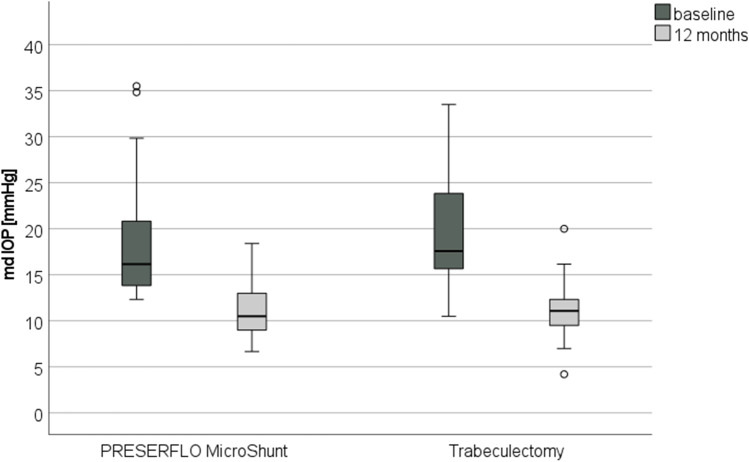
Boxplots showing mean diurnal intraocular pressure (mdIOP) at baseline and at the 12-month follow-up in the MicroShunt and trabeculectomy group. * Statistical significance (P < 0.05, Wilcoxon test)

One year after surgery (Table 2), median [Q25, Q75] mdIOP (Fig. 1) decreased statistically significantly from maximally treated 16.2 [13.8–21.5] to 10.5 [8.9–13.5] mmHg without medication (*p* < 0.001, Wilcoxon test) in the MicroShunt and from 17.6 [15.6–24.0] to 11.1 [9.5–12.3] mmHg without medication (*p* < 0.001, Wilcoxon test) in the trabeculectomy group. Absolute (MicroShunt: -6.3 [-11.3 – (-4.2)] mmHg; trabeculectomy: -7.5 [-14.2—(-4.0)] mmHg; *p* = 0.60, Mann–Whitney *U* test) and relative IOP reduction (MicroShunt: -40.4 [-51.7 – (-27.3)] %; trabeculectomy: -43.9 [-59.1—(-26.5)] %; *p* = 0.79, Mann–Whitney *U* test) were not statistically significantly different between the 2 groups. Median peak diurnal IOP [Q25, Q75] decreased from 22.0 [17.0–27.5] to 13 [12–16.0] mmHg (*p* < 0.001, Wilcoxon test) in the MicroShunt and from 23.5 [20.0–30.0] to 14.0 [12.5–15.3] mmHg (*p* < 0.001, Wilcoxon test) in the trabeculectomy group. Median [Q25, Q75] diurnal IOP fluctuation was also statistically significantly reduced in both groups (Table 2; all *p* < 0.001, Wilcoxon test).

**Table 2:** Median diurnal IOP, peak IOP, IOP fluctuation, medication use, BCVA, visual fields and endothelial cell counts at 12 months

	PRESERFLO™ MicroShunt			Trabeculectomy			
	Baseline	12 months	*p* value^~^	Baseline	12 months	*p* value^~^	*p* value*
n	30	29		30	30		
mdIOP [mmHg]	16.2 [13.8—21.5]	10.5 [8.9—13.5]	** < 0.001**	17.6 [15.6—24.0]	11.1 [9.5—12.3]	** < 0.001**	-
mdIOP reduction [mmHg]		-6.3 [-11.3 – (-4.2)]	-		-7.5 [-14.2 – (-4.0)]	-	0.596
mdIOP reduction (%)		-40.4 [-51.7 – (-27.3)]			-43.9 [-59.1 – (-26.5)]		0.785
peak of dIOP [mmHg]	22.0 [17.0—27.5]	13.0 [12.0—16.0]	** < 0.001**	23.5 [20.0—30.0]	14.0 [12.0—15.3]	** < 0.001**	-
Reduction diurnal peak IOP [mmHg]		-8.0 [-14.0 – (-4.0)]			-10.0 [-17.7 – (-3.7)]	-	0.702
Reduction diurnal peak IOP (%)		-34.8 [-54.8 – (-24.3)]			-43.4 [-59.4 – (-18.8)]	-	0.903
fluctuation of dIOP [mmHg]	10.0 [6.0—12.5]	5.0 [3.5—6.0]	** < 0.001**	8.5 [5.0—13.3]	5.0 [4.0—7.0]	**0.002**	-
Reduction of diurnal IOP fluctuation [mmHg]		-4.0 [-8.0 – (-0.5)]	-		-3.5 [-7.7—0.3]	-	0.528
Reduction of diurnal IOP fluctuation (%)		-45.5 [-69.7 – (-8.3)]	-		-41.4 [-67.4—5.6]	-	0.413
no. IOP-lowering medications	4 [3 – 4]	0 [0 – 0]	** < 0.001**	4 [4 – 4]	0 [0 – 0]	** < 0.001**	-
BCVA [LogMar]	0.05 [0.00—0.10]	0.07 [0.00—0.14]	0.311	0.15 [0.05—0.30]	0.18 [0.10—0.30]	0.259	-
MD of VF [dB]	-5.7 ([-11.9 – (-3.0)]	-4.2 [-8.3 – (-1.9)]	**0.002**	-9.1 [-19.2 – (-6.5)]	-11.3 [-16.6 – (-7.7)]	0.604	-
EC [cells/mm^2^]	2566 [2239 – 2788]	2471 [2201 – 2785]	0.737	2149 [1880 – 2365]	2074 [1782 – 2485]	0.463	-

IOP = intraocular pressure; BCVA = best corrected visual acuity; mdIOP = mean diurnal intraocular pressure; MD = mean deviation; EC = endothelial cell count

Presented as median and interquartile range IQR [Q25 – Q75]

^*^ Mann–Whitney-U test; ~ Wilcoxon test

**Fig. 2:**
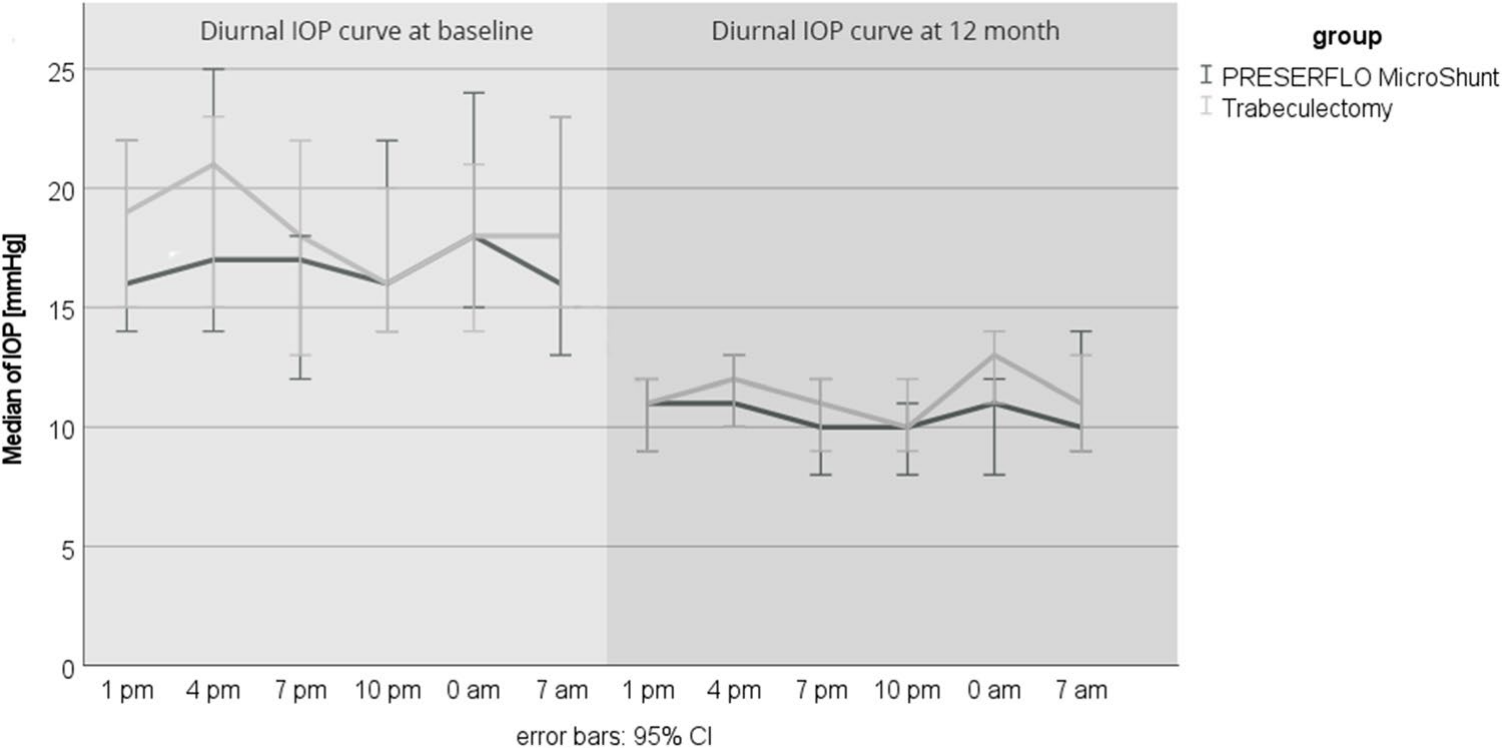
Graph showing the median diurnal intraocular pressure (IOP) at the different measurement times at baseline and at 12 months in the MicroShunt and trabeculectomy group. CI, confidence interval

Median [Q25, Q75] diurnal IOPs at the different measurement times at baseline and at 1 year of both procedures are shown in Fig. 2.

### Secondary outcome measures

**Fig. 3:**
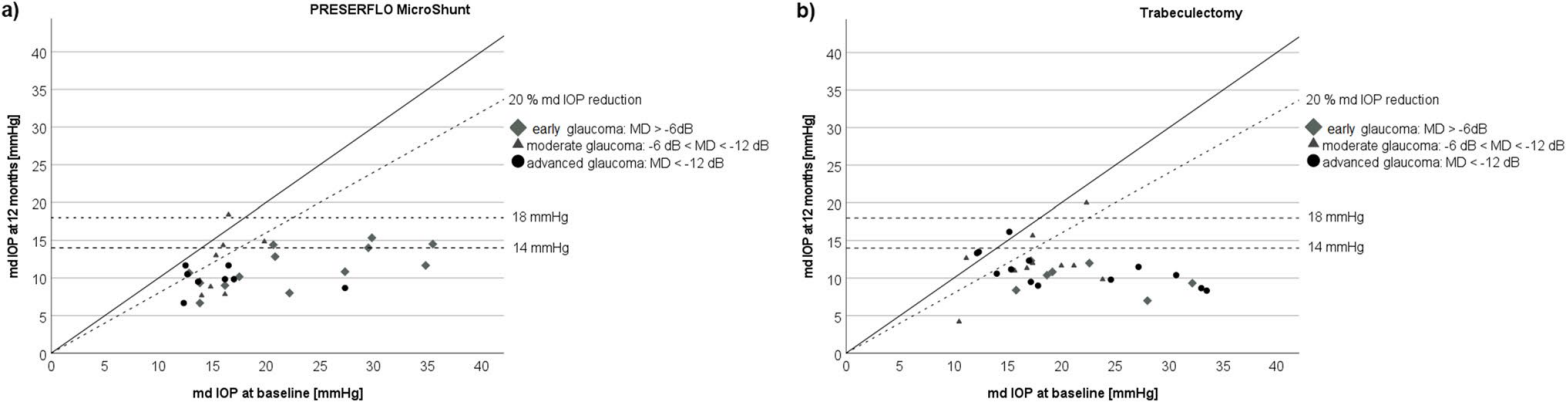
**a**) Scatterplot showing mean diurnal intraocular pressure (mdIOP) reductions at 12 months ≤ 18 and ≤ 14 mmHg as well as ≤ 20% in the MicroShunt group. **b**) Scatterplot showing mean diurnal intraocular pressure (mdIOP) reductions at 12 months ≤ 18 and ≤ 14 mmHg as well as ≤ 20% in the trabeculectomy group

**Fig. 4:**
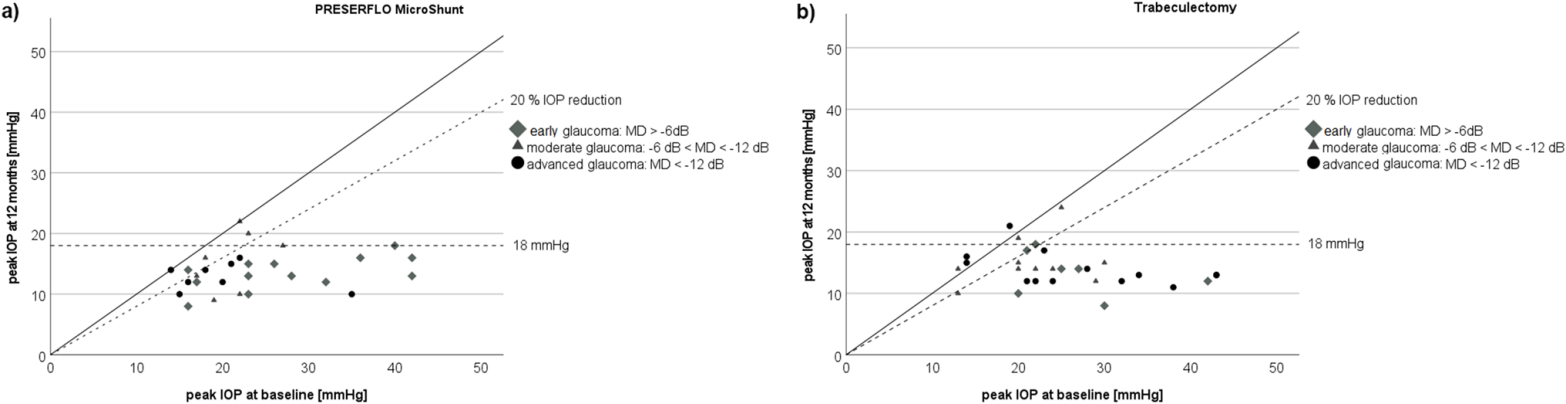
**a**) Scatterplot showing mean diurnal peak intraocular pressure reductions at 12 months ≤ 18 as well as ≤ 20% in the MicroShunt group. **b**) Scatterplot showing mean diurnal peak intraocular pressure reductions at 12 months ≤ 18 as well as ≤ 20% in the trabeculectomy group

Success and failure rates are shown in Table 3. One hundred per cent of eyes with early glaucoma and without threat of fixation, of which 11 were in the MicroShunt and 5 in the trabeculectomy group, fulfilled the criteria for complete success (mdIOP and diurnal peak IOP ≤ 18 mmHg without hypotony or the need of any IOP-lowering medication) in both groups (*p* = 1.0, Fisher exact test, Fig. 3 and 4). Eyes with early glaucoma, with threat of fixation and with moderate to advanced glaucoma, of which 19 were in the MicroShunt and 25 in the trabeculectomy group, fulfilled complete success criteria (mdIOP ≤ 14 mmHg and diurnal peak IOP ≤ 18 mmHg without hypotony or the need of IOP-lowering medication) in 74% of cases in the MicroShunt and in 88% in the trabeculectomy group (*p* = 0.26, Fisher exact test, Fig. 3 and 4). One patient (5%) in the MicroShunt group showed qualified success, needing 1 IOP-lowering medication. Overall, success was 87% in both groups.

**Table 3:** Success and failure rates

	PRESERFLO™ MicroShunt (*n* = 30)	Trabeculectomy (*n* = 30)	*p* value^+^
No. (%) of eyes below ≤ 18 mmHg without meds	28 (93)	29 (97)	1.0
No. (%) of eyes below ≤ 14 mmHg without meds	23 (77)	27 (90)	0.335
No. (%) of eyes with ≥ 20% mdIOP reduction	24 (80)	24 (80)	1.0
No. (%) of eyes below ≤ 18 mmHg of peakIOP	28 (93)	27 (90)	1.0
No. (%) of eyes with ≥ 20% peakIOP reduction	25 (83)	22 (73)	0.532
No. (%) Complete success ^a^)	11 (100)	5 (100)	1.0
No. (%) Complete success ^b^)	14 (74)	21 (88)	0.467
No. (%) Qualified success ^b^)	1 (5)	0 (0)	0.432
No. (%) Overall success ^a+b^)	26 (87)	26 (87)	1.0
No. (%) Failure	4 (13)	4 (13)	1.0
Surgical	1 (3)	0	
Pressure	3 (10)	4 (13)	

^a^Early cases without threat of fixation and ≤ 18 mmHg, *n* = 11 in the PRESERFLO™ MicroShunt group, *n* = 5 in the trabeculectomy group ^b^ early cases with threat of fixation and moderate and advanced cases ≤ 14 mmHg, *n* = 19 in the PRESERFLO™ MicroShunt group, *n* = 25 in the trabeculectomy group. ^+^ Fisher exact test

According to the definition for failure in the current study there were 4 (13%) failures in each group (Table 3). One (3%) surgical failure due to bleb scarring and 3 pressure failures in the MicroShunt group vs 4 pressure failures in the trabeculectomy group. Only 1 patient of the cases with pressure failures in each group was receiving anti-glaucoma medication again, which was reintroduced at the ophthalmologists´ discretion. The IOP at 1 year was presumably considered to be satisfactory in the absence of progression in the other cases. In the MicroShunt group there was no pressure failure due to an IOP ≤ 5 mmHg whereas there was one such case in the trabeculectomy group. There was no failure due to loss of light perception.

The number of IOP-lowering medications decreased statistically significantly in both groups (*p* < 0.001, Wilcoxon test). Best spectacle-corrected visual acuity (MicroShunt group: *p* = 0.31; trabeculectomy group: *p* = 0.26; Wilcoxon test) and endothelial cell density (MicroShunt group: *p* = 0.74; trabeculectomy group: *p* = 0.46; Wilcoxon test) did not change significantly at 1 year after surgery, in both groups. Visual field defects ameliorated in the Microshunt group (*p* = 0.002, Wilcoxon test) and stayed stable in the trabeculectomy group (*p* = 0.6, Wilcoxon test) (all Table 2).

### Postoperative interventions and adverse events

Complications and interventions during the early postoperative period (within 4 weeks), between 4 weeks to 6 months, and 6 to 12 months are summarized in Table 4. The most prominent complication in the early postoperative period was a transient postoperative hypotony causing choroidal effusion—an abnormal accumulation of fluid in the suprachoroidal space [28], which required AC stabilization in 8 cases (27%) of PRESERFLO™ MicroShunts and 7 cases (23%) of trabeculectomies. Early bleb encapsulation requiring bleb needling occurred in 1 case (3%) of the PRESERFLO™ MicroShunt and in 4 cases (13%) of the trabeculectomy group. Complications such as a Seidel positive leakage, hyphema or the need for laser suture lysis occurred only in the trabeculectomy group. Between 4 weeks and 6 months no interventions were needed in the PRESERFLO™ MicroShunt group, whereas in the trabeculectomy group, 9 interventions (3 laser suture lysis, 6 bleb needlings) were needed. Between 6 and 12 months, 3 interventions (2 bleb needlings, 1 bleb revision) were needed in the PRESERFLO™ MicroShunt group and 5 interventions (1 surgical suture because of leakage, 1 laser suturolysis, 1 AC reformation, 1 bleb needling, 1 phacoemulsification) in the trabeculectomy group.

**Table 4:** Complications and interventions within 4 weeks, between 4 weeks and 6 months and between 6 and 12 months postoperatively

	PRESERFLO™ MicroShunt	Trabeculectomy	*p* value
Complications and interventions within 4 weeks	*n* (%)	*n* (%)	
- Seidel positive leakage	0 (0)	6 (20)	**0.023**
- Hypotony ≤ 5 mmHg at any time	16 (53)	9 (30)	0.115
- Hypotony requiring AC reformation	8 (27)	7 (23)	0.776
- 1 AC reformation	6 (20)	2 (7)	0.254
- 2 AC reformations	2 (7)	2 (7)	1.0
- 3 AC reformations	0 (0)	2 (7)	0.491
- > 3 AC reformations	0 (0)	1 (3)	1.0
- Choroidal effusion	8 (27)	7 (23)	0.776
- Hypotony maculopathy	0 (0)	1 (3)	1.0
- Laser suture lysis	0 (0)	13 (43)	** < 0.001**
- Flat anterior chamber	0 (0)	0 (0)	1.0
- hyphema	0 (0)	1 (3)	1.0
- encapsulation + bleb needling	1 (3)	4 (13)	0.353
Complications and interventions between 4 weeks and 6 months			
- Seidel positive leakage	0 (0)	0 (0)	1.0
- Laser suture lysis	0 (0)	3 (10)	0.237
- encapsulation + bleb needling	0 (0)	6 (20)	**0.023**
- fibrosis + bleb revision	0 (0)	0 (0)	1.0
- prolonged hypotony + AC stabilization	0 (0)	1 (3)	1.0
- increased cataract formation + phacoemulsification	0 (0)	0 (0)	1.0
- secondary IOP lowering surgery	0 (0)	0 (0)	1.0
Complications and interventions between 6 and 12 months			
- Seidel positive leakage	0 (0)	1 (3)	1.0
- Laser suture lysis	0 (0)	1 (3)	1.0
- encapsulation + bleb needling	2 (7)	1 (3)	1.0
- fibrosis + bleb revision	1 (3)	0 (0)	1.0
- prolonged hypotony + AC stabilization	0 (0)	1 (3)	1.0
- increased cataract formation + phacoemulsification	0 (0)	1 (3)	1.0
- secondary IOP lowering surgery	0 (0)	0 (0)	1.0
Severe adverse events			
- blebitis or endophthalmitis	0 (0)	0 (0)	1.0
- corneal decompensation	0 (0)	0 (0)	1.0
- retinal detachment	0 (0)	0 (0)	1.0
- suprachoroidal hemorrhage	0 (0)	0 (0)	1.0
- malignant glaucoma	0 (0)	0 (0)	1.0
- no light perception	0 (0)	0 (0)	1.0
- microshunt erosion	0 (0)	n.a	-
Total no. of patients with interventions (no. of interventions)	8 (14)	18 (51)	**0.018**

AC = anterior chamber; IOP = intraocular pressure; no. = number

Data are presented as n = number (% = percentage). Fisher exact test; significance is marked in bold (*p* 4 < 0.05)

In summary, during the first year eight patients needed 14 interventions in the PRESERFLO™ MicroShunt (10 AC reformations, 3 bleb needlings, 1 bleb revision) and 18 patients needed 51 interventions in the trabeculectomy group (21 AC reformations, 17 laser suture lysis, 11 bleb needlings, 1 surgical suture because of leakage, 1 phacoemulsification), which was statistically significantly different (*P* = 0.018, Fisher exact test). No severe adverse events such as blebitis, endophthalmitis, corneal decompensation, retinal detachment, suprachoroidal haemorrhage, malignant glaucoma or loss of light perception were seen. There were no device migrations or erosions in the MicroShunt group.

## Discussion

The current study presents the 1-year data evaluation of the efficacy and safety of a stand-alone primary PRESERFLO™ MicroShunt implantation compared to a stand-alone primary trabeculectomy, both with adjunctive MMC (0.02% for 3 min) and intracameral injection of 3 mg Bevacizumab (Avastin®, Roche, USA) [24] in similar study cohorts of White/European patients with POAG. Besides being of the same genetic/ethnic background, patients of both groups were matched for age, known duration of disease, and number and classes of IOP-lowering medication to have a comparable conjunctival condition, which is essential for the outcome of filtration surgery. This was possible because all patients are part of the Dresden Glaucoma and Treatment Study (DGTS).

As already observed in the 6-month analysis [17], both procedures continued to be equally effective in lowering mean and peak diurnal IOP as well as IOP fluctuations and glaucoma medications over one year. The postoperative management was more intense in the trabeculectomy group, especially in the early postoperative period with statistically more patients needing interventions [17, 29]. Although the rate of patients needing AC reformation in the early postoperative period was not statistically significantly different between both groups, 8 patients in the MicroShunt and 7 patients in the trabeculectomy group (*p* = 0.776), only 2 patients in the MicroShunt group needed a second AC reformation. MicroShunt patients recovered rather quickly from a transient hypotony. In the trabeculectomy group 5 patients needed multiple AC reformations due to overfiltration. Overfiltration in the MicroShunt group could be prevented by inserting a 10–0 nylon thread through the shunt which could be removed as needed, this was not carried out in the current study. Overfiltration in the trabeculectomy group could be prevented by suturing the scleral flap with additional 10–0 nylon sutures. In the current study only 2 sutures were used to fixate the scleral flap in all cases to make the procedure more comparable. Despite these rather frequent AC reformations in the early postoperative period, mainly in the first 4 weeks postoperatively, outcomes at 1 year were excellent. In both groups there were no serious adverse events, such as blebitis, endophthalmitis, corneal decompensation, retinal detachment, suprachoroidal haemorrhage, malignant glaucoma or loss of light perception occurring during the first year after surgery. There were no device migrations, erosions or damaging effects on the endothelium seen in the MicroShunt group. Of course follow-up of 1 year might be too short to find these possible complications.

Interestingly visual field defects decreased statistically significantly in the MicroShunt group and stayed stable in the trabeculectomy group. Reasons for this might be the better visual fields at baseline in the MicroShunt group with maybe a better benefit from complete cessation of topical glaucoma therapy [30] and/or due to a learning effect.

A recently published prospective, randomized, multicenter, noninferiority study by Baker et al. [2] conducted in the United States and Europe also compared the effectiveness and safety of the MicroShunt (*n* = 395) versus trabeculectomy (*n* = 132) in patients with POAG one year after surgery. The probability of success was statistically significantly lower in the MicroShunt group compared with trabeculectomy (53.9% vs. 72.7%, respectively; *p* < 0.01).

**Fig. 5:**
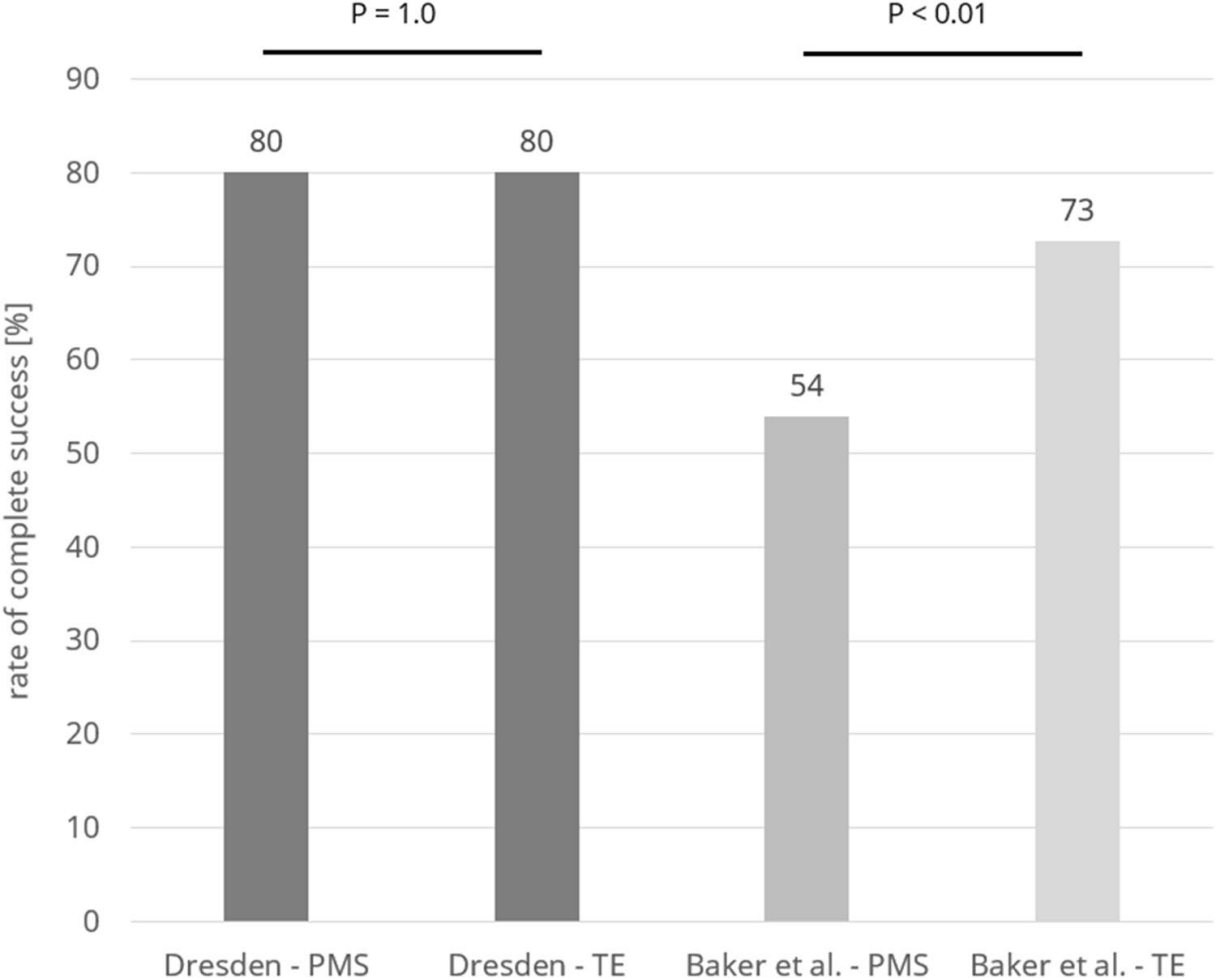
Comparison of the rate of complete success according to the definitions of the study by Baker et al. [2] which is a ≥ 20% IOP reduction from baseline at 1 year without increasing the number of glaucoma medications between the current study and the study by Baker et al. PMS = PRESERFLO MicroShunt; TE = trabeculectomy

Applying the same success criteria used in the study by Baker et al. which was defined as a ≥ 20% IOP reduction from baseline at 1 year without increasing the number of glaucoma medications, 80% of the patients in the current study who had received the MicroShunt and 80% of the patients who had received a trabeculectomy fulfilled these criteria (Fig. 5). Contrary to the study by Baker et al. where success rates were significantly lower with the MicroShunt (53.9% vs 72.7%; *p* < 0.01) there was no difference in success rates between both groups in the present study (*p* = 1.0; Fisher exact test).

Mean IOP (mean of 3 measurements at 9:00am ± 1.5 h, 12:00 pm ± 1 h, 4:00 pm ± 2 h) decreased from 21.1 ± 4.9 mmHg to 14.3 ± 4.3 mmHg with a mean of 0.6 ± 1.1 glaucoma medications at year 1 in the MicroShunt and from 21.1 ± 5.0 mmHg to 11.1 ± 4.3 mmHg with a mean of 0.3 ± 0.9 glaucoma medications in the trabeculectomy group, in the study by Baker et al.

In the present study median mdIOP (mean of 6 measurements including one in the supine position at 7:00am ± 0.5 h, 1:00 pm ± 0.5 h, 4:00 pm ± 0.5 h, 7:00 pm ± 0.5 h, 10:00 pm ± 0.5 h, 00:00am ± 0.5 h) decreased from 16.2 [13.8–21.5] to 10.5 [8.9–13.5] mmHg without medication (*p* < 0.001, Wilcoxon test) in the MicroShunt and from 17.6 [15.6–24.0] to 11.1 [9.5–12.3] mmHg without medication (*p* < 0.001, Wilcoxon test) in the trabeculectomy group. Absolute and relative IOP reduction were not statistically significantly different between the 2 groups (Table 2).

One of the reasons for these differing results might be the higher proportion of Black/African Americans in the MicroShunt group compared with the trabeculectomy group (18.0% vs. 8.3%; *p* < 0.01) in the study by Baker et al. Maybe ancestrally different glaucoma patients have different conjunctival conditions with different wound healing, risk of bleb fibrosis and bleb failure [31]. A European study on the safety and efficacy of the MicroShunt found that non-Caucasian ethnicity was the only risk factor consistently associated with increased failure [32]. Another reason might be, that although patients did not have a history of conjunctival surgery, angle or trabecular meshwork surgery 6 months prior to study entry was allowed, whereas the cases in the current study were all first-time glaucoma surgeries. It is not clear whether there might have been a difference between the groups in the study by Baker et al. regarding prior glaucoma surgeries. Finally the study was conducted across 29 sites with 58 surgeons having little experience with the MicroShunt and greater experience with trabeculectomy. Although this is also the case in the current single center study, only 2 surgeons were involved, resulting in an increase of individual experience with the rather new device more rapidly. Moreover both procedures were standardized in the current study, especially regarding MMC use, whereas the study by Baker et al. allowed for some flexibility with MMC use.

As in the study by Baker et al., the current study confirms that endothelial cell loss was not statistically significant and similar in both groups at 1 year, which is an encouraging finding.

The study by Baker et al. found that the rate of complications and interventions was much less after MicroShunt implantation, particularly in the early postoperative period. This is in concordance with the current study and our previous study [17]. As a consequence, this resulted in less frequent postoperative visits in the MicroShunt group and a more predictable postoperative course which might not only be beneficial for the patient´s quality of life but also timesaving for the surgeon and cost saving for the medical system.

A recent 2-year, prospective, single-arm, multicenter study by Beckers et al. [15] on the safety and effectiveness of the PRESERFLO™ MicroShunt on 81 patients with POAG showed an IOP decrease from 21.7 ± 3.4 (*n* = 81) to 14.5 ± 4.6 (*n* = 67) at year 1 and 14.1 ± 3.2 mmHg (*n* = 60) at year 2 (*P* < 0.0001). Overall success, which was defined as not requiring reoperation, an IOP ≤ 21 mmHg and ≥ 20% reduction in IOP with and without supplemental glaucoma medication use, at year 1 (*n* = 67) was 74.1%. Although baseline IOP of the MicroShunt group in the current study was much lower (median mdIOP 16.2 [13.8–21.5] mmHg) the IOP at year 1 was also lower (10.5 [8.9–13.5] mmHg), even without medication (*p* < 0.001, Wilcoxon test). Using the success criteria of this multicenter study, 80% of the patients in the current study fulfilled these criteria at year 1, despite the rather low IOP at baseline. Taking into account that only 67 patients were evaluated at year 1 in the multicenter study, overall success is only 62%.

The authors found a trend towards better IOP reduction and statistically significant less glaucoma medications in a 0.4 mg/ml MMC group compared to a 0.2 mg/ml MMC group. The current study only used 0.2 mg/ml MMC since all cases were first-time incisional glaucoma surgeries with IOP still being lower at year 1 (10.5 [8.9–13.5] mmHg) compared to the 0.4 mg/ml MMC group in the multicenter study (13.7 ± 3.4 mmHg).

Reasons might be the inclusion of different ethnicities, the inclusion of juvenile glaucoma (the youngest participant was 28 years), varying surgical techniques and MMC concentrations as well as postoperative management (choice between bleb needling and revision) among the sites [7] and surgeons [11].

There is some discussion whether a higher MMC concentration (0.4 mg/ml) may contribute to a better IOP-lowering efficacy and reduction of glaucoma medications [2, 15, 26]. The results of the current study indicate that 0.2 mg/ml MMC is sufficient in first-time incisional glaucoma surgery of POAG patients even in advanced cases and with a glaucoma medical history of on average 10 years. A greater MMC concentration with a higher risk for adverse events should probably not be used as a routine in first-time incisional surgery of POAG patients.

Another study by Wagner et al. [21], which assessed surgical success between XEN45® gelstent, PRESERFLO™ MicroShunt and trabeculectomy with MMC had a rate of strict success (i.e. IOP ≤ 18 mmHg and > 5 mmHg and a 20% IOP reduction) of 64.7% in the trabeculectomy, 31.4% in the XEN and 54.8% in the MicroShunt group 6 months after surgery. This was statistically significantly different (*p* = 0.02) between the three groups, but not between the trabeculectomy and MicroShunt group (*p* = 0.42). Although 1-year data cannot be compared with 6-month data, the current study shows a “strict” success rate of 80% in both groups, which confirms that both procedures are equally effective in lowering IOP, also at 1 year. The reason for the better outcome in the current study is probably the inclusion of only patients with POAG with a comparable conjunctival condition.

The longest study on the MicroShunt to date is the feasibility study by Batlle et al. [16, 33] presenting data of 23 eyes of Hispanic patients with POAG not at target with maximum tolerated IOP-lowering medication, who underwent MicroShunt implantation augmented with 0.4 mg/ml MMC, and were followed for 3 years [33] as well as in an extension study, for 5 years [16]. Their results showed a mean reduction of IOP from 23.8 ± 5.3 mmHg to 10.7 ± 3.5 (*n* = 22) three and 12.4 ± 6.5 mmHg (*n* = 21) five years after surgery. At 1 year, the average IOP in patients who received the MicroShunt in a standalone procedure (*n* = 14; 60%) was about 11.3 mmHg, which is quite similar to our 1-year results.

A retrospective interventional case series by Schlenker et al. [26] on 164 eyes of 132 patients with a broad mix of baseline characteristics found 76.9% of eyes with complete (without glaucoma medication) and 92.5% with qualified (with glaucoma medication) success (no 2 consecutive IOP readings > 17 mmHg or clinical hypotony and at least a 20% reduction from decision IOP) one year after PRESERFLO™ MicroShunt implantation. Although the group was quite inhomogeneous, with only 53% of Caucasian ethnicity and only 67% suffering from POAG, complete success was similar to the current study (80% with the above success criteria).

A retrospective multicenter study by Tanner et al. [34] on the real-world efficacy and safety of the PRESERFLO™ MicroShunt using MMC 0.4 mg/ml shows a complete success rate (IOP of 6–21 mmHg and a 20% reduction) of only 51.9% after one year, with a failure rate of 31.7%. Using these criteria, this compares to 80% complete success and 3% failure (1 surgical failure, all pressure failures were below 21 mmHg) rates in the current study. Reasons for the differences are mainly the mix of different ethnicities, glaucoma diagnoses, and high number of previous ocular and glaucoma surgeries.

Variations in the results of PRESERFLO™ MicroShunt studies may be attributed to differences in demographics with different ethnicities, glaucoma diagnoses and preoperative conditions. Also the number, expertise, and surgical techniques of the surgeons may play a role. Finally, different criteria for surgical endpoints render comparisons difficult.

The implantation of the MicroShunt is less invasive and less traumatic to the eye relative to trabeculectomy. There is no scleral flap, no peripheral iridectomy or tension sutures, which reduce the occurrence of hyphemas in the AC, inflammation, bleb procedures and the need for laser suture lysis. The better predictability encourages to recommend incisional glaucoma surgery earlier in the treatment paradigm of glaucoma. This is an important aspect regarding the disadvantages of long-term glaucoma medication which can not only damage the corneal epithelium [30] but also the conjunctiva so that treatment success of filtration surgery is limited [35].

### Limitations

The current study has several limitations. Follow-up time and number of cases included are still rather short. The sample size is limited due to the strict inclusion and exclusion criteria as well as matching the groups for age, duration of application, number and classes of IOP-lowering medications. This way both surgical groups were rather homogenous however. The study group intends to continue evaluating surgical outcomes after 2- and 5-years follow-up.

PRESERFLO™ MicroShunt patients who met the inclusion and exclusion criteria were consecutively included, whereas trabeculectomy patients were not. This might have caused a selection bias. But that way, in order to assume a similar conjunctival condition as close as possible preoperatively, the trabeculectomy group could be age-matched and had the same exposure to IOP-lowering medications, which is important when comparing filtration surgery.

The results represent treatment outcomes of White/European patients with POAG and cannot offer conclusions on effectiveness and safety in other ethnic groups or glaucoma entities.

All surgeries were performed by 2 surgeons, which makes it less generalizable.

### Strengths

The study population was homogenous in regard to ethnicity, glaucoma diagnosis and age. The exposure to medical therapy was the same, which allows for the assumption of compareable preoperative conjunctival conditions, since healing might be different otherwise. Both groups were part of the Dresden Glaucoma and Treatment Study using the same study design, with the same inclusion and exclusion criteria as well as standardized definitions of success and failure, postoperative treatment and follow-up. Important strengths of the study are the evaluation of standardized diurnal IOP measurements, including a measurement in the supine position, rather than comparing IOP measurements taken at non-standardized points in time. Also, the inclusion of patients with relatively low baseline IOP is a strength of this study.

## Conclusions

In conclusion, the present study demonstrates similar and sustained surgical success and safety of the PRESERFLO™ MicroShunt compared with trabeculectomy, one year after surgery in POAG patients with moderate to advanced disease in need of a low IOP. It confirms once more that the MicroShunt has the potential to be an alternative to trabeculectomy, the current gold-standard surgical treatment for these patients. The less invasive approach with a higher predictability, especially in the early postoperative period [17], makes postoperative care less time consuming, with less visits and infrequent interventions needed and probably encourages to recommend incisional glaucoma surgery earlier. This is particularly advantageous in times of pandemics such as the Covid-19 pandemic [36]. The more posterior bleb might be beneficial in regard to possible long-term complications such as blebitis and bleb-related endophthalmitis, which can occur years after trabeculectomy [5]. This increases the patient´s quality of life, reduces medication burden and might save medical costs or at least balance the higher initial costs.

## Data Availability

Data is available on request.
